# Effectiveness of a Chatbot-Based Internet-Support-Coping-Caring Intervention in Improving Sleep Quality and Work-Related Outcomes Among Nurses in Taiwan During the COVID-19 Pandemic: A Pilot Study

**DOI:** 10.1097/jnr.0000000000000691

**Published:** 2025-08-04

**Authors:** Hui-Ling LAI, Chun-I CHEN, Guan-Hsiung LIAW, Liu-Chun LU, Chiung-Yu HUANG

**Affiliations:** 1 Nursing Department, Tzu Chi University, Hualien, Taiwan, ROC; 2 Bachelor Program in Interdisciplinary Studies, National Chung Cheng University, Chiayi, Taiwan, ROC; 3 Information Engineering Department, I-Shou University, Kaohsiung, Taiwan, ROC; 4 Operating Room, E-DA DaChang Hospital, Kaohsiung, Taiwan, ROC; 5 Nursing Department, I-Shou University, Kaohsiung, Taiwan, ROC; † Contributed equally

**Keywords:** anxiety and depressive symptoms, perceived stress, sleep quality, Internet-support-coping-caring With Chatbot

## Abstract

**Background:**

Work-related stress has long been a significant concern in the nursing profession. The pressures faced by nurses in the current health care climate have been further exacerbated by the COVID-19 pandemic. Interventions using chatbot to support nurses may help improve nurse health during the pandemic. Given the rising health care costs and the current crisis, exploring and developing innovative interventions to support nurses and promote their health are essential.

**Purpose:**

This research was designed to investigate the effectiveness of the Internet-Support-Coping-Caring with Chatbot (ISCC Chatbot) on sleep quality, perceived stress, anxiety, and depressive symptoms among nurses in Taiwan. Measurements were taken at baseline (T1) and at 3-week (T2) and 6-week (T3) follow-ups.

**Methods:**

An experimental research design was used. To minimize bias, eligible participants who met the inclusion criteria were assigned randomly to either the intervention or control group (*n* = 40 per group). The intervention group received 6 weeks of the ISCC Chatbot intervention, while the control group was placed on a waiting list and received regular care. A general estimation equation was used to analyze the impact of the intervention on perceived stress, sleep quality, anxiety, and depressive symptoms.

**Results:**

At T2, perceived stress and depressive symptom scores (with the exception of anxiety and sleep quality) were significantly better in the intervention group than the control group. At T3, perceived stress, sleep quality, anxiety, and depressive symptom scores were all significantly better in the intervention group than the control group. While the intervention group improved significantly in sleep quality, perceived stress, and anxiety between T1 and T2 and between T2 and T3, the control group improved significantly in sleep quality and depressive symptoms only between T1 and T2. At T3, sleep quality, perceived stress, and depressive symptoms in the control group had significantly worsened.

**Conclusions/Implications for Practice:**

Based on the results, combining ISCC with a chatbot provide to supervisors a potentially effective approach to enhancing nurse health. Participants noted the efficacy of the chatbot, particularly when working alone, in reducing work-related stress. Integrating ISCC Chatbot into the nursing workplace is recommended to provide support and safeguard the welfare of nurses, particularly during challenging times.

## Introduction

The serious global impact of the COVID-19 pandemic between 2019 and 2023 presented a widespread threat to public health. Despite the development of effective vaccines, challenges such as emerging variants, vaccine hesitancy, supply chain disruptions, and limited access to health care have persisted. As we transition into the postpandemic era, coronavirus variants continue to affect health care workers, leading to elevated levels of depression, anxiety, and stress (Karabulak & Kaya, 2021; Zhu et al., 2020). The COVID-19 pandemic has significantly impacted the sleep quality and mental health of nurses, with studies showing nurses experiencing poorer sleep quality and higher levels of anxiety than the general population (Çürük, et al., 2023; Sampaio et al., 2023; Sikaras et al., 2023). During the pandemic, around 35% of health care workers have experienced depression, 16% have experienced anxiety, and 15.9% have faced other psychological stressors (Liu et al., 2020). The pandemic has regularly placed nursing professionals, especially clinical nursing staff, in intensely stressful conditions (Karabulak & Kaya, 2021), for example, grief over losing colleagues to COVID-19, heavy workloads, emergency shifts, and staffing shortages (Siga Tage et al., 2023). High stress levels have negatively affected the sleep quality and mental health of nurses, which in turn have affected their work and endangered patient safety (Sedano et al., 2023).

During the pandemic, health care workers have experienced exceptionally high levels of work-related stress, leading to sleep and mental health problems. However, social distancing protocols made face-to-face psychological counseling impractical. With advancements in internet technologies and the rapid development of artificial intelligence in recent years, chatbots have been increasingly utilized in research to assess and intervene in mental health. Hungerbuehler et al. (2021) discovered a chatbot-based workplace mental health assessment to be a highly engaging and effective tool for collecting anonymized mental health data from employees. Anmella et al. (2023) found chatbots to be valuable in screening for and assessing the severity of anxiety and depressive symptoms as well as for detecting suicide risk. Yosep et al. (2023) found digital-based nursing interventions, including mobile and web-based approaches, to improve mental health in adolescents by reducing stress, anxiety, and depression, while enhancing resilience, well-being, and self-efficacy. Despite the evidence provided by these studies, there is a notable absence of research on the use of AI-powered chatbots to ameliorate sleep, stress, anxiety, and depression in health care workers. This study was motivated by the need to address this research gap.

Evidence in the literature supports that relaxing music can reduce cortisol levels and stress in health care professionals (Chang et al., 2018; Lai & Li, 2011). Music has been found to affect nurses’ mental health positively by inducing psychological stability, reducing feelings of embitterment, and increasing psychological well-being (Choi & Choo, 2023). Moreover, spending more time listening to music and engaging in more musical activities can further enhance psychological stability in nurses (Wang et al., 2022). While exercise has numerous established health benefits, including improved cardiorespiratory fitness, muscular fitness, and bone health, evidence in the literature suggests that a large percentage of nurses do not engage in sufficient physical activity (Owusu-Sekyere, 2020). One pilot study found that participating in yoga sessions positively impacted perceived stress levels and muscle fatigue in nurses (Anderson et al., 2017). In light of evidence that exercises such as yoga promote improvements in mental health, sleep quality, and perceived stress in nurses, both music and exercise components were integrated into the proposed intervention program.

Limitations on research resources and access during the COVID-19 outbreak have encouraged the use of cross-sectional studies (Karabulak & Kaya, 2021). Although Taiwan’s rapid control measures have helped keep nurses safe and healthy (Chen et al., 2020), ongoing mutations emphasize the necessity for further interventions to maintain nurse health. COVID-19 stress has significantly affected nurses. Despite Taiwan’s current success in controlling the COVID-19 outbreak domestically, nursing staff continue to face substantial pressures. Thus, this study was designed to investigate the effect on nurse stress and health of a mobile app-based intervention support program using generalized estimating equations to analyze changes over time. The goal of this pilot study was to develop an innovative methodology to reduce stress levels in nurses, which, in turn, may be expected to enhance their quality of sleep and decrease anxiety and depressive symptoms.

## Methods

### Design

This study employed an experimental research design from August 2021 to May 2022. Convenience sampling was used to recruit and enroll 80 eligible participants, all of whom were nurses at a hospital in southern Taiwan. Recruitment was conducted via intranet email announcements. To promote fairness, eligible nurses meeting the inclusion criteria were assigned randomly to either the intervention or control group (*n*=40 per group). The intervention group received 6 weeks of the Internet-Support-Coping-Caring With Chatbot (ISCC Chatbot) intervention, while the control group received a standard intervention that included either relaxation or counseling and was placed on a waiting list. Strategies to minimize the risk of intergroup contamination between participants working in the same unit included blinding, education, monitoring, and enforcement (Armijo-Olivo et al., 2022). Blinding minimizes participant information sharing, while educating participants on the importance of confidentiality discourages the sharing of details. Ongoing monitoring ensures protocol adherence and prompt intervention, with confidentiality agreements helping maintain research integrity. The installation of the ISCC Chatbot on the personal phones of intervention group members further minimized the risk of participants sharing private content.

### Participants

**Figure 1:**
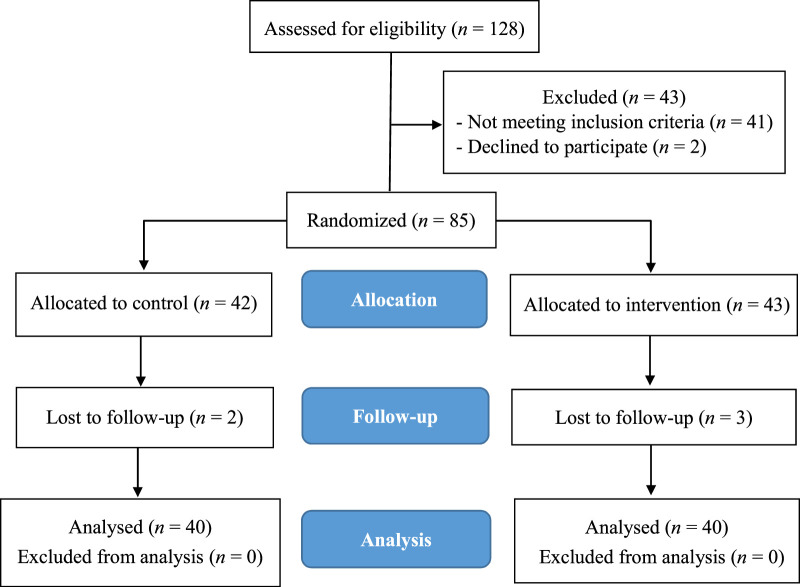
Flow Diagram of the Internet-Support-Coping-Caring Chatbot Intervention *Note.* Intervention was initiated in the experimental group with Internet-Support-Coping-Caring Chatbot. Control group patients received only usual care from a head nurse throughout the study period. Outcomes (Perceived stress, sleep quality, anxiety, depressive symptoms) were measured at T1, T2, and T3.

A minimum sample size calculation using Cohen’s (1988) *G* Power 3.1 was conducted assuming a moderate effect size of 0.25, a significance level of α=.05, and a desired power of 1-β=0.80. The study used a repeated-measures statistical analysis method, and a target of at least 70 eligible participants was set with an additional 15% buffer to compensate for potential withdrawals. The inclusion criteria were the following: 20 years of age or older, at least 3 months of nursing experience, and scoring either above 5 on the Pittsburgh Sleep Quality Index (PSQI) or above 7 on the Hospital Anxiety and Depression Scale (HADS). Nurses who did not meet the inclusion criteria, did not have a smartphone or tablet, or did not regularly access the internet were excluded. The Consolidated Standards of Reporting Trials guidelines (Moher et al., 2001) were followed in this research. The enrolled participants were randomized into intervention and control groups using a randomized single-blind controlled trial design. The flow diagram of this study is given in Figure 1. Of the 128 nurses screened for the primary study between August 2021 and March 2022, 87 met the inclusion criteria and 41 did not. Two subsequently declined to participate, and 43 were allocated to the intervention group and 42 were allocated to the control group. The intervention was completed in May 2022. During the follow-up period, two participants in the control group and three in the intervention group were lost to follow-up. Thus, valid data from 80 participants (40 in the intervention and 40 in the control) were available for analysis. The respective attrition rates were 7% and 4.8% in the intervention and control groups.

Perceived stress, sleep quality, anxiety, and depressive symptoms were assessed at baseline (T1) and at 3 weeks (T2) and 6 weeks (T3) follow-up.

### Measures

Measures included demographic information, work characteristics, sleep quality, and perceived stress, anxiety, and depressive symptoms. Each is described in the following.

#### Demographic Information and Work Characteristics

The demographic questionnaire collected information on age, marital status, education, income, professional ranking, work experience, and work unit.

#### Sleep Quality

The PSQI was developed by Buysse and colleagues (1989) to assess sleep quality using 19 items under seven components coverings sleep duration, disturbances, medication use, and other aspects. The total possible score range is 0 to 21, with higher scores indicating worse sleep quality. Scores above 5 suggest inadequate sleep, while those at 5 or below signify adequate sleep. The internal consistency (Cronbach’s α) of the PSQI was estimated to be .80 (Huang et al., 2023) and was .78 in this study.

#### Perceived Stress

Perceived stress was assessed using the 14-item Chinese version of Cohen and colleagues' Perceived Stress Scale (1983). Items are assessed on a 5-point Likert scale (ranging from 0 to 56), with higher scores indicating higher perceived stress. The scale includes 7 positive (Q4, Q5, Q6, Q7, Q9, Q10, Q13) and 7 negative (Q1, Q2, Q3, Q8, Q11, Q12, Q14) items. The Cronbach’s alpha of the original (Cohen et al., 1983) scale ranged from .84 to .86, with a test-retest reliability of .85. In this study, the Cronbach’s alpha coefficient of the Chinese version was .83.

#### Anxiety and Depressive Symptoms

The HADS was developed by Zigmond and Snaith (1983) to assess disease-related anxiety and depression symptoms. The 14 items of the scale are divided into 7 for the anxiety (HAD-A) and 7 for the depression (HAD-D) subscales, with each item scored from 0 to 3. Each subscale has a total score range of 0–21, with higher scores indicating more severe symptoms. Scores under 7 are interpreted as normal, 8–10 indicate mild symptoms, 11–14 indicate moderate symptoms, and 15–21 indicate severe symptoms. The HADS has been assessed as having strong internal consistency with a Cronbach’s alpha of .82 for anxiety and .79 for depression (Lai et al., 2020). In this study, Cronbach’s α values were .81 for the HADS-Anxiety subscale and .83 for the HADS-Depression subscale.

#### Intervention

The intervention group was given access to the ISCC Chatbot app for 6 weeks, and the effectiveness of the ISCC Chatbot in improving the sleep quality, perceived stress, and mental health of users was assessed based on Lazarus and Folkman’s stress, appraisal, and coping theory (Lazarus & Folkman, 1984) and Miller and Rollnick’s (1991) approach to cognitive-behavioral education. The ISCC Chatbot is designed to provide care support, knowledge enhancement, and skill building.

The intervention supports group and individual discussions, messages, and relaxation techniques via an interactive chatbot app with elements of generative pretrained transformer technology. The goal of this study was to assess the effectiveness of this intervention in terms of reducing perceived stress, enhancing sleep quality, and improving mental health in nurses.

**Figure 2:**
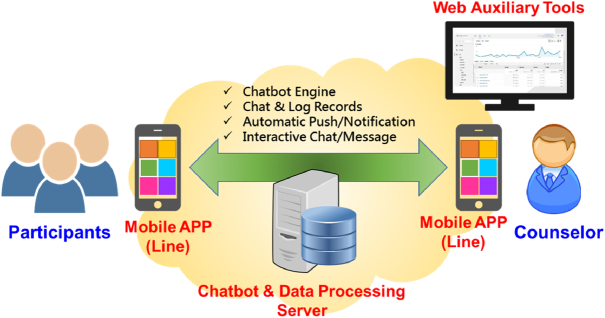
Operational architecture of the Internet-Support-Coping-Caring Chatbot System

The operational architecture of the ISCC Chatbot system is illustrated in Figure 2. This system offers the following web-based tools to the administrator (facilitator): (a) chatbot, which automatically responds to chat messages from the participants’ app based on ISCC Chatbot content; (b) data upload, which enables the case or administrator to upload personal information, questionnaire answers, and meeting records to the server’s database storage; and (c) message exchange, which facilitates interactions, conversations, and messaging between the administrator (facilitator) and participants via the ISCC Chatbot mobile app. The ISCC Chatbot app includes (a) personal information—a space in which users can write their personal thoughts and record situational experiences; (b) chatbot with elements of generative pretrained transformer technology—providing interactive care; and (c) activities—consisting of relaxation skill training (video) and exercise (video) content.

Regular ISCC Chatbot operations include the following: (a) weekly interactions between the participants and research coordinator over the 6-week intervention and the uploading of daily logs including their feedback, relaxation and exercise activities, and engagement in these activities at least three times a week and (b) the provision of supportive content, including automated reminders to listen to music, daily support messages from the principal investigator, and chatbot messages. The program was designed to encourage participant engagement through regular interaction with the research coordinator and the provision of supportive content. A detailed description of the intervention program is given below:

1. Weeks 1–3
  The first half of the intervention (week 1–3) was designed to improve participants’ daily behaviors and promote sleep hygiene for better psychological and physical health. The researchers explained the importance of sleep hygiene, provided educational information accessible through the app, and taught the participants how to maintain a sleep diary and monitoring chart. The intervention also emphasized the beneficial effects of exercise on sleep, eating a balanced diet, keeping regular meal times, consuming sleep-inducing foods, and avoiding stimulants (such as caffeine, nicotine, and alcohol) before bedtime.
  To monitor progress, participant sleep records and monitoring charts were collected weekly, and any questions from the previous week were addressed by the facilitator. The importance of sleep was reinforced, and participants were advised to avoid repeatedly checking the clock while trying to sleep, establish regular bedtimes, and use the recommended bedroom equipment to improve sleep quality.
  This part of the intervention aimed to provide the participants with the practical knowledge and tools necessary to establish healthy sleep habits and promote better physical and psychological health.
2. Weeks 4–6

The second half of the intervention (weeks 4–6) was designed to teach the participants presleep mental control techniques such as relaxation skills and the avoidance of stimulating activities. The researchers assessed participant skills, suggested ways to increase proactive coping strategies, and selected appropriate problem-solving methods. Effective stress and emotional management techniques were also provided.

The sleep part of the intervention included “sleep-restriction activities” designed to calculate sleep efficiency and limit the time spent in bed as an aid to cultivating healthier sleep patterns and enhancing overall sleep quality. Sleep logs and monitoring data were collected from participants for the first three weeks and used to reinforce the importance of regulating the sleep–wake cycle and to encourage their continued application of sleep hygiene rules as a non-pharmacological therapy to enhance sleep quality.

Multidimensional support measures were employed to ameliorate participants’ perceived stress and potentially reduce the prevalence of sleep disorders. Information on stress management, emotional regulation, and psychological distress was provided, and sleep-promoting audiovisual materials were used to further reinforce sleep-related knowledge and self-control efficacy. The app facilitated the communication of sleep-promoting strategies, and participants learned techniques to enhance relaxation skills, promote calming thoughts, and avoid stimulating activities. Thus, the goal of the second half of the intervention was to teach practical strategies that participants could continue to apply in managing stress, improving mental health, and enhancing sleep quality.

### Ethical Considerations

The study protocol was approved (EMRP23110N) by the institutional review board of a hospital in southern Taiwan. Written informed consent was obtained from all of the participants prior to data collection.

### Statistical Analysis

Statistical analyses were conducted in line with the research objectives using the SPSS Predictive Analytics Software package (PASW Statistics 23), and *p* values below .05 were interpreted to reflect statistical significance. The collected data were subjected to normality testing to confirm normal distribution, and all potential interaction effects were controlled. Descriptive and inferential statistics, including independent *t* tests and a Generalized Estimating Equation (GEE), were applied. GEE was used for both within- and between-group longitudinal data analyses. The dependent variables were: perceived stress, sleep quality, anxiety, and depressive symptoms. Data were collected at baseline (T1) and at 3-week (T2) and 6-week (T3) follow-ups.

## Results

### Participant Characteristics

Characteristics data for the 80 participants (40 intervention and 40 control), including age, marital status, educational level, income, seniority, work unit, and professional experience, were collected and analyzed (Table 1). The mean age was 33.6 (*SD*=6.87) years, over half were single, and 83.8% (67) held a bachelor’s degree or above. In terms of income, 20% earned an individual monthly income below NTD 40,000, and 80% earned an income above this amount. Most (*n*=33, 41.2%) held the lowest-level position for nursing professionals, i.e., N1. Most nurses worked in outpatient departments (*n*=48, 60%), followed by operating rooms (*n*=22, 27.5%), and wards (*n*=10, 12.5%). The mean duration of professional experience was 10.42 months (*SD*=6.82). All of the participants were female. Notably, no between-group differences in the demographic or job characteristics were identified.

**Table 1:** Between-group Comparison of Demographic and Work Characteristics (*N*=80).

Characteristic	*n* (%)		Total	*p*
	Control Group	Experimental		
	(*n*=40)	(*n*=40)		
Age (year; mean±*SD*)	33.15±7.47	34.10±6.27	33.60±6.87	.541
Marital status				.644
Single	25 (62.5)	27 (67.5)	52 (65.0)	
Married (cohabit)	15 (37.5)	13 (32.5)	28 (35.0)	
Educational level				.258
Junior college	9 (22.5)	4 (10.0)	13 (16.2)	
Bachelor or above	31 (77.5)	36 (90.0)	67 (83.8)	
Income (NTD; monthly)				.322
<40,000	8 (20.0)	8 (20.0)	16 (20.0)	
40,000–80,000	19 (47.5)	25 (62.5)	44 (55.0)	
>80,000	13 (32.5)	7 (17.5)	20 (25.0)	
Professional Rank				.283
N1	14 (35.0)	19 (47.5)	33 (41.2)	
N2	19 (47.5)	17 (42.5)	36 (45.0)	
N3 or N4	7 (17.5)	4 (10.0)	11 (13.8)	
Work experience				.695
(months; mean±*SD*)	10.12±7.14	10.73±6.57	10.42±6.82	
Unit				.820
Ward	4 (10.0)	6 (15.0)	10 (12.5)	
Operating room	15 (37.5)	7 (17.5)	22 (27.5)	
Outpatient department	21 (52.5)	27 (67.5)	48 (60.0)	

*Note.* NTD = New Taiwan Dollars.

### Between and Within-group Differences in Outcome Variables at Different Time Points

#### Sleep Quality (Sleep Disturbances)

**Figure 3:**
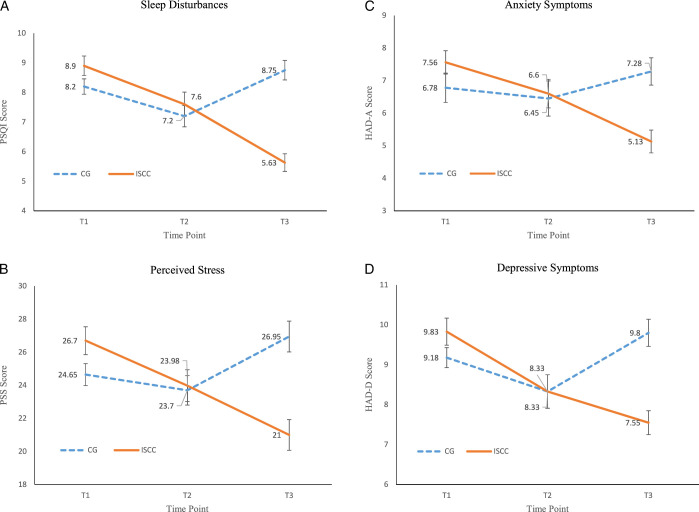
Outcome Variables Comparison Between Control and Intervention Groups at 3 Different Time Points (T1, T2 and T3) *Note.* PSQI=Pittsburgh Sleep Quality Index; PSS=Perceived Stress Scale; HAD-A=Hospital Anxiety and Depression-Anxiety Scale; HAD-D=Hospital Anxiety and Depression-Depression Scale.

As shown in Table 2 and Figure 3, the primary analysis of sleep disturbances was conducted using GEE statistical analysis. No significant difference in sleep disturbances was found between the intervention and control groups at baseline (β=−0.70, *p*=.097). However, the intervention group reported progressively lower disturbances at T2 and T3 than the control group, with the intergroup difference at T2 not reaching statistical significance (β=−0.30, *p*=.331) and that at T3 reaching statistical significance (β=−3.80, *p*<.001).

**Table 2:** Distribution of Outcome Variables at Pretest (T1) and at 3 (T2) and 6 Weeks (T3) and Between/Within-group Comparisons (*N*=80).

Predictor Variable	Control Group (*n*=40)				Intervention Group (ISCC; *n*=40)				Between Group ^a^		
	Mean (*SE*)	β	Within Group ^b^	*p*	Mean (*SE*)	β	Within Group ^c^	*p*	β	95% CI	*p*
			95% CI				95% CI				
Sleep disturbances											
T1	8.20 (0.26)	Ref.			8.90 (0.33)	Ref.			Ref.		
T2	7.20 (0.36)	−1.00	[−1.47, −0.53]	<.001	7.60 (0.41)	−1.30	[−1.68, −2.84]	<.001	−0.30	[−0.91, 0.31]	.331
T3	8.75 (0.33)	0.53	[0.08, 0.97]	.022	5.63 (0.30)	−3.28	[−3.70, −0.92]	<.001	−3.80	[−4.20, −3.18]	<.001
Perceived stress											
T1	24.65 (0.66)	Ref.			26.70 (0.84)	Ref.			Ref.		
T2	23.70 (0.89)	2.30	[−2.28, 0.38]	.162	23.98 (0.96)	−2.70	[−3.69, −1.70]	<.001	−1.75	[−3.41, −0.09]	.039
T3	26.95 (0.93)	−0.95	[0.97, 3.63]	.001	21.00 (0.93)	−5.68	[−6.76, −4.59]	<.001	−7.98	[−9.69, −6.26]	<.001
Anxiety symptoms											
T1	6.78 (0.45)	Ref.			7.56 (0.36)	Ref.			Ref.		
T2	6.45 (0.54)	−0.33	[−1.41, 0.76]	.558	6.60 (0.44)	−0.95	[−1.55, −0.35]	.002	−0.63	[−1.87, 0.62]	.324
T3	7.28 (0.42)	0.50	[−0.28, 1.28]	.210	5.13 (0.35)	−2.43	[−3.09, −1.76]	<.001	−2.93	[−3.95, −1.90]	<.001
Depressive symptoms											
T1	9.18 (0.25)	Ref.			9.83 (0.34)	Ref.			Ref.		
T2	8.33 (0.42)	−0.85	[−1.49, −0.21]	.009	8.33 (0.42)	−0.08	[−0.44, 0.29]	.691	0.78	[0.04, 1.51]	0.39
T3	9.80 (0.34)	0.63	[0.15, 1.10]	.010	7.55 (0.30)	−2.28	[−2.78, −1.77]	<.001	−2.90	[−3.60, −2.20]	<.001

*Note.* ISCC = Internet-Support-Coping-Caring; T1 = baseline; T2 = ISCC Chatbot 3rd week; T3 = ISCC Chatbot 6th week; Ref. = reference group.

^a^ The differences between groups result from the (group × time effect) of GEE. T2 is an additional change in scores for the ISCC Chatbot intervention group compared with the usual care group at T2; T3 is defined as an additional change in scores for the ISCC Chatbot intervention group compared with the usual care group at T3.

^b^ The within-group differences result from changes in the usual care group over time, using the generalized estimating equation (GEE) to analyze. T2 is defined as a change of scores for the usual care group at T2 compared with T1; T3 is defined as a change of scores for the usual care group at T3 compared with T1.

^c^ The within-group differences result from changes in the intervention group over time, using the GEE to analyze. T2 is defined as a change in scores for the intervention group at T2 compared with T1; T3 is defined as a change of scores for the ISCC Chatbot intervention group at T3 compared with T1.

*p<.05. **p<01. ***p<.001.

As shown in Figure 3A, a significant reduction in sleep disturbances (from 8.9 at T1 to 5.63 at T3) was reported for the intervention group, while that in the control group increased slightly over the same period (from 8.2 at T1 to 8.75 at T3).

#### Perceived Stress

As shown in Table 2 and Figure 3B, GEE statistical analysis was used in the primary analysis of perceived stress. No significant between-group difference in perceived stress was identified at baseline (β=2.03, *p*=.059). However, the intervention group reported progressively lower perceived stress at T2 and T3 than the control group, with the intergroup differences at T2 and T3 both reaching statistical significance (β=−1.75, *p*=.039 and β=−7.98, *p*<.001, respectively).

As shown in Figure 3B, a significant (5.7 points) decline in perceived stress level (from 26.7 at T1 to 21 at T3) was reported for the intervention group, while that in the control group increased modestly (2.30 points) over the same period (from 24.65 at T1 to 26.95 at T3).

#### Anxiety Symptoms

As shown in Table 2 and Figure 3C, GEE statistical analysis was used in the primary analysis of anxiety. No significant difference in anxiety was found between the intervention and control groups at baseline (β=0.78, *p*=.178). However, the intervention group reported progressively lower levels of anxiety at T2 and T3 than the control group, with the intergroup difference at T2 not reaching statistical significance (β=−0.63, *p*=.324) and that at T3 reaching statistical significance (β=−2.93, *p*<.001).

As illustrated in Figure 3C, a consistent decline in anxiety symptoms was observed, with the ISCC Chatbot group decreasing from 7.56 (T1) to 5.13 (T3), indicating a reduction of 2.43 points, while the control group experienced a slight increase from 6.78 (T1) to 7.28 (T3), reflecting a 0.50-point increment.

#### Depressive Symptoms

As shown in Table 2 and Figure 3D, GEE statistical analysis was used in the primary analysis of depressive symptom severity. No significant between-group difference in depressive symptom severity was identified at baseline (β=0.65, *p*=.097). However, the intervention group reported progressively lower depressive symptoms at T2 and T3 than the control group, with the intergroup differences at T2 and T3 both reaching statistical significance (β=0.78, *p*=.039 and β=−2.90, *p*<.001, respectively).

As illustrated in Figure 3D, a significant (2.28 points) decline in depressive symptom severity (from 9.83 at T1 to 7.55 at T3) was reported for the intervention group, while that in the control group increased slightly (0.62 point) over the same period (from 9.18 at T1 to 9.8 at T3).

## Discussion

This study was conducted to evaluate the impact of an ISCC Chatbot intervention on sleep quality, stress, anxiety and depression in nurses during a professionally difficult period of time (i.e., the COVID-19 pandemic). The findings reveal discernible disparities in sleep quality between intervention and control groups. Although the intervention group exhibited enhanced sleep quality, the magnitude of the between-group change at T2 did not reach statistical significance, suggesting the need for a longer intervention period or application of alternative therapies to achieve statistically significant effects. Notably, the significant between-group difference at T3 indicates the intervention may require a longer implementation period to meaningfully improve sleep quality. Also, the intervention may be further refined to promote prolonged engagement and optimize the overall impact.

In terms of perceived stress, significant improvements in the intervention group were reported at both T2 and T3, with statistically significant between-group differences observed at both time points. These findings suggest the intervention positively and significantly impacts perceived stress levels. As this is a new intervention, no previous studies are available for comparison. However, the results are consistent with other studies on nursing work-related stress. For example, Lai and Li (2011) observed music therapy and relaxation interventions to positively influence physiological stress indicators in nurses.

The intervention in this study was also shown to be effective in reducing anxiety symptom severity, even after accounting for time and group interactions. The percentage change between T2 and baseline T1 was higher, but not significantly so, in the intervention group than in the control group. Notably, the percentage change between T3 and T1 was significantly greater in the intervention group, indicating a more significant effect of the intervention over time. The intergroup differences at T2 and T3 were both significantly different, supporting a significant reduction effect of the ISCC Chatbot app on depressive symptoms.

Regarding the impact of the intervention on stress, anxiety, depressive symptoms, and sleep disturbances, the results demonstrated significant improvements in sleep quality, work-related stress, and overall mental health in the intervention group at both T2 and T3. Interestingly, both the intervention and control groups showed improvements in sleep quality, stress, anxiety, and depressive symptoms at T2. However, only the intervention group sustained these benefits through T3, indicating a more enduring effect. Nurses in the control group faced constraints such as demanding schedules and were often required to leave their posts for supportive counseling. These limitations, coupled with increased workloads due to the COVID-19 pandemic and ongoing staff shortages, likely contributed to heightened anxiety and occasional lapses in attention.

Because nursing managers were only able to offer supportive counseling during working hours, the control group may have benefited initially from these efforts, particularly at T2, where improvements in sleep disturbances and mental health were observed. These gains were likely driven by early-stage counseling sessions facilitated by head nurses. However, ongoing workplace demands may have limited the overall effectiveness of these interventions. As the situation worsened by T3, nurses in the control group experienced a deterioration in sleep quality and mental health, potentially exacerbated by the lack of access to flexible support resources like the ISCC Chatbot. In contrast, nurses in the intervention group were able to engage with the ISCC Chatbot at their own convenience, allowing them to manage responsibilities more flexibly and effectively. The chatbot served as a readily accessible platform for stress relief, available anytime and anywhere, which may explain why this group maintained their improvements through T3-despite similar outcomes between both groups at T2.

It is also important to highlight that the decline in mental health among control group nurses may be attributed to the chronic stress and heavy workloads commonly faced in the nursing profession. Future research should examine the factors underlying such negative outcomes and focus on developing targeted interventions. Overall, these findings suggest that flexible, technology-assisted support systems may be particularly beneficial for improving sleep quality, reducing occupational stress, and managing anxiety and depression among nurses.

### Limitations

The potential limitations of this study include the following: (1) Generalizability: this study was conducted at a specific working moment for nurses during the COVID-19 pandemic, which may limit the generalizability of the findings to other health care settings and populations. (2) Unpredictability: another limitation is the ongoing and unpredictable nature of the pandemic, which may have affected the motivation of participants to fully comply with ISSC program components such as the exercise or relaxation sections in the mobile app, potentially influencing the results. (3) Work characteristics: diverse work characteristics can result in varying levels of work stress and load. However, due to the small sample size, the effects of different work characteristics were not explored and should be investigated in future research.

To address these limitations, future studies should consider developing more appealing and effective strategies to encourage participants to comply with intervention protocols. Moreover, the follow-up period was relatively short (only 6 weeks), which may have limited the ability to assess the long-term effectiveness of the intervention.

Future research should address these limitations by controlling for potential confounding variables, explicitly examining the impact of the pandemic on mental health outcomes, and increasing the follow-up period to assess the long-term effectiveness of the intervention. Overall, although this study provides valuable insights on the potential benefits of an ISCC Chatbot intervention for improving the mental health of nurses during the pandemic, the findings should be interpreted with caution and further research is needed to confirm and extend these results.

### Conclusions

The results of this study support the potential effectiveness of an ISSC program combined with a chatbot intervention in improving sleep and mental health outcomes for nurses during pandemics and other stress-inducing medical events. The results indicate the intervention group showed a greater reduction in anxiety and depressive symptoms, improved sleep quality, and lower work stress than the control group. Although this study has some limitations, the findings provide valuable insights into the potential benefits of utilizing technology-based interventions to promote mental health among health care professionals.

### Implications for Nursing Practice

The findings of this study have significant implications for nursing practice. Nurses play a critical role in the health care system, and their mental health is essential to providing high-quality care to patients. The results suggest that nurses can benefit from technology-based interventions to improve their mental health outcomes, particularly during the COVID-19 pandemic and similar events that impose high levels of stress on health care professionals. Therefore, nursing educators and administrators should consider incorporating technology-based interventions into their training programs to equip nurses with the necessary tools to cope with their job demands. Moreover, this study highlights the importance of prioritizing nurses’ mental health in the workplace. Nursing leaders and policymakers should create supportive work environments that promote nurses’ mental health, including access to mental health resources, regular mental health assessments, and training programs to improve their coping skills. By prioritizing nurses’ mental health, health care organizations can improve job satisfaction, reduce burnout, and enhance the quality of care provided to patients. Finally, further research is required to confirm and extend these results, including studies with larger sample sizes, longer follow-up periods, and more diverse populations.
